# Improving Health Worker Adherence to Malaria Treatment Guidelines in Papua New Guinea: Feasibility and Acceptability of a Text Message Reminder Service

**DOI:** 10.1371/journal.pone.0076578

**Published:** 2013-10-07

**Authors:** Serah F. Kurumop, Chris Bullen, Robyn Whittaker, Inoni Betuela, Manuel W. Hetzel, Justin Pulford

**Affiliations:** 1 Papua New Guinea Institute of Medical Research (PNGIMR), Goroka, Eastern Highlands Province, Papua New Guinea; 2 National Institute for Health Innovation, School of Population Health, The University of Auckland, Auckland, New Zealand; 3 Swiss Tropical and Public Health Institute, Basel, Switzerland; 4 University of Basel, Basel, Switzerland; 5 School of Population Health, The University of Queensland, Herston, Queensland, Australia; University of Massachusetts Medical School, United States of America

## Abstract

The aim of this study is to assess whether a text message reminder service designed to support health worker adherence to a revised malaria treatment protocol is feasible and acceptable in Papua New Guinea (PNG). The study took place in six purposively selected health facilities located in the Eastern Highlands Province (EHP) of PNG. Ten text messages designed to remind participants of key elements of the new NMTP were transmitted to 42 health workers twice over a two week period (two text messages per day, Monday to Friday) via the country’s largest mobile network provider. The feasibility and acceptability of the text message reminder service was assessed by transmission reports, participant diaries and group discussions. Findings indicate that the vast majority of text messages were successfully transmitted, participants’ had regular mobile phone access and that most text messages were read most of the time and were considered both acceptable and clinically useful. Nevertheless, the study found that PNG health workers may tire of the service if the same messages are repeated too many times and that health workers may be reluctant to utilize more comprehensive, yet complementary, resources. In conclusion, a text message reminder service to support health worker adherence to the new malaria treatment protocol is feasible and acceptable in PNG. A rigorous pragmatic, effectiveness trial would be justified on the basis of these findings.

## Introduction

Papua New Guinea (PNG), a malaria endemic nation of approximately seven million people, introduced a revised national malaria treatment protocol (NMTP) in late 2011. Consistent with current WHO guidelines [1], the new NMTP stipulates that all fever or suspected malaria cases be tested for malaria infection by microscopy or rapid diagnostic test (RDT) and that anti-malarials should only be prescribed upon confirmation of malaria parasitaemia [2]. This represents a substantial change in recommended malaria case management as, prior to the new NMTP, the presumptive treatment of fever patients with anti-malarials was a near universal practice [3]. The revised NMTP also introduced artemether-lumefantrine (AL) as the first line treatment for uncomplicated malaria in place of chloroquine, amodiaquine and sulphadoxine-pyrimethamine, drugs to which a high degree of parasite resistance has developed [4-6]. An AL and primaquine combination is further recommended in cases of *P. vivax* infection, as this malaria species is widespread throughout PNG [7]. Thus, the new NMTP not only requires PNG health workers to adopt a more rigorous process when diagnosing and treating malaria, but also prescribe medications to which they have had little if any prior exposure (AL) [3,8] or with potentially harmful side effects (primaquine) [9].

The experiences of other countries that have similarly revised their respective malaria treatment protocols suggest health worker adherence may be problematic. For example, three years after the implementation of a protocol stipulating routine microscopy or RDT testing of all adult fever cases and the prescription of AL to test positive cases in Kenya, testing rates in health facilities with RDT or microscopy available did not exceed 54% and nearly a third of test negative cases were prescribed AL [10]. A recent review noted that health worker adherence to revised antimalarial prescription protocols generally improves over time [11], yet few studies included in the review reported adherence rates higher than 70% even several years after protocol implementation and many reported adherence rates lower than 50%.

Those studies that have evaluated interventions designed to improve adherence to malaria treatment guidelines have reported mixed success. A six day integrated management of malaria course designed to encourage communication and trust among various health workers involved in treating febrile patients in Uganda produced sustained improvements in some aspects of clinical practice 12 months post intervention, but not all [12]. The impact of a three-day in-service training and the provision of various resources and job aids on malaria case management were modest at best in a pre-/post-intervention study conducted in Kenya [13]. The authors subsequently concluded that one-off training interventions, even when supported by training materials and job aids, are unlikely to be effective if follow-up support and supervision is not provided. An earlier review paper noted that adherence to long-standing (as opposed to recently introduced) malaria treatment protocols was often poor if recent quality improvement interventions had not been implemented [14], further suggesting continuous support and supervision is required to maintain high performance standards. A review of the broader health care literature similarly concluded that multiple means of support are almost always necessary to improve health worker performance [15].

Reflecting the benefit of regular longer-term support, a recent Kenyan study demonstrated a substantial improvement in health worker adherence to malaria treatment guidelines via the provision of regular text message reminders [16]. Ten discrete text messages, each describing a recommended malaria case management practice, were variously sent to participating health workers personal mobile phones twice a day, five days a week over a six-month period. A 24.5% improvement in malaria case management practice (based on adherence to national treatment guidelines) was subsequently observed in the intervention group *versus* the control group six months post intervention. Furthermore, the intervention was favourably compared to other forms of training and support by participating health workers [17] and was shown to be highly cost effective [18]. These findings contribute to the emerging evidence-base indicating that simple interventions delivered via mobile communication technology can improve health care service delivery [19].

The PNG National Department of Health aimed to train all health workers in the new NMTP prior to implementation and a number of supportive job aids such as RDT user guides and AL prescription posters were widely disseminated. Nevertheless, the substantial change in clinical practice that the new NMTP requires as well as the evidence indicating high rates of protocol adherence necessitates multiple and continuous forms of health worker support suggests further interventions may be needed. A text message reminder service of the type described above has potential applicability to PNG where health workers are often working in isolation, far from sources of support and supervision. Furthermore, the rapid growth of mobile phone ownership from around 5% in 2007 to almost 40% in 2011 combined with widespread mobile network coverage of 70% (Oxford Business Group Report http://www.oxfordbusinessgroup.com/economic_updates/papua-new-guinea-banking-great-unbanked, Dec 3 2012) in PNG offers a hitherto unavailable opportunity to deliver interventions that might in part address the impact of geographical and cost barriers to sustained adoption of adherence to protocols. Accordingly, the aim of this study is to assess whether a text message reminder service designed to complement existing means of NMTP support, is feasible and acceptable to health workers in PNG. Key research questions included: Is the mobile phone network stable enough to ensure reliable text message transmission? Do health workers have regular enough access to their mobile phones to ensure intervention exposure? Are the proposed text messages easily understood by PNG health workers? Do PNG health workers consider the text message reminder service an acceptable and useful form of support?

## Methods

### Study Population

The study took place in six purposively selected health facilities located in the Eastern Highlands Province (EHP) of PNG. Two of the health facilities were located in an urban centre and four in rural locations across EHP. Health facilities were considered eligible for inclusion if they: employed five or more health workers, were located in an area with mobile phone coverage, and if a majority of the respective health workers owned their own mobile phone. Health workers were considered eligible for inclusion if they owned or had reliable access to a mobile phone on the target network and if they treated malaria patients as part of their clinical duties.

### Procedures

The 10 text messages (listed in Table 1) were designed to remind participants of key elements of the new NMTP. The message content was developed by the authors in collaboration with the PNG national malaria technical working group. The text messages were pre-tested for comprehension with health workers not involved in the study. All text messages were written in English, as this is the language typically employed in health worker training and support materials in PNG, and were limited to a maximum of 160 characters. Once developed, the text messages were provided to a gateway service operated through the National Institute of Health Innovation, University of Auckland, New Zealand. The gateway provider was responsible for transmitting the text messages according to an automated schedule via the PNG Digicel network to all participating health workers. The gateway provider was selected for cost and convenience reasons, operating from the same institute as two of the co-investigators (CB and RW). The Digicel network was chosen as it is the largest in PNG, although at least two other network providers were in operation at the time of this study (B-Mobile and Citifon). Two text messages were transmitted each day (at 9.00 am and 2.00pm, respectively) between Monday and Friday, ensuring all ten messages were sent over a five day period (schedule is listed in Panel 1). This transmission schedule was repeated twice over consecutive weeks during the trial.

**Table 1:** Text message content and transmission schedule.

**Message 1, Monday morning:**
Test ALL fever or suspected malaria patients using RDT or mps, even if patient is less than 5 years old.
**Message 2, Monday afternoon:**
Explain the purpose of the RDT/mps test to the patient. Advise the patient what a positive or negative test result indicates.
**Message 3, Tuesday morning:**
Do NOT prescribe any anti-malarial to patients with a negative RDT/mps test.
**Message 4, Tuesday afternoon:**
Advise ALL patients with a negative RDT/mps test to return to the health facility in 3 days if fever persists or symptoms worsen.
**Message 5, Wednesday morning:**
Record the RDT/mps results daily in the National Health Information System (NHIS) Outpatient Tally Sheet and the Health Facility Malaria Register.
**Message 6, Wednesday afternoon:**
Advise ALL fever or suspected malaria patients to sleep under an insecticide treated mosquito net EVERY night of the year.
**Message 7, Thursday morning:**
Prescribe Mala-1 to ALL patients with a positive RDT/mps test, unless patient is in first trimester of pregnancy.
**Message 8, Thursday afternoon:**
Advise patient or their caregiver to complete 6 doses of Mala-1 tablets over 3 days, even if the patient feels better after just a few doses.
**Message 9, Friday morning:**
Prescribe primaquine if RDT/mps test result confirms non-*P. falciparum* infection, *P. vivax* infection or mixed *P. falciparum* infection.
**Message 10, Friday afternoon:**
Advise patients to stop taking primaquine if their urine becomes a darker colour and to return to the health facility immediately.

RDT = Rapid Diagnostic Test, mps = malaria parasite slide, Mala-1 = brand of artemether-lumefantrine.

A scientific officer visited each prospective health facility to assess whether the inclusion criteria were met in consultation with the respective officer in charge. If inclusion criteria were met, then the scientific officer provided a written and verbal explanation of the trial to all eligible health workers and sought signed consent to participate. The two week text message trial commenced the Monday following study site selection. Transmissions across the six study sites were completed between November and December, 2012.

The feasibility of the text message reminder service was assessed by transmission reports from the gateway provider specifying whether the scheduled text messages were successfully delivered to each of the mobile numbers provided. In addition, participants were asked to record daily entries in a diary provided by the research team. Daily diary questions included: whether the participant’s designated mobile phone (i.e. the phone to which the text messages were being sent) was available to them that day; times and reasons for mobile phone unavailability (if unavailability reported); whether the participant received any text messages that day; which messages were received; the times at which they were received and the times at which they were read. Acceptability was assessed via group discussions (GD) with participating health workers at the conclusion of the two week trial. The GDs were conducted by a scientific officer, were held at each of the participating health facilities with as many participants as were available and were typically 30-60 minutes in length. All GDs were guided by an interview schedule that canvassed: mobile network coverage, mobile phone access, text message access, text message content, frequency and timing of text message transmission, and the perceived utility of the text message reminder trial.

### Ethics statement

All participants provided written informed consent. This study was approved and granted ethical clearance by the Medical Research Advisory Committee of Papua New Guinea (MRAC No.12.18; 5 July 2012). No incentives of any kind were offered to study participants.

### Complementary data

To support the feasibility component of this pilot study, two questions pertaining to mobile phone ownership (‘do you own a mobile phone?’) and network membership (‘if yes, what network(s) do you belong to?’) were included in the health worker interview component of a country wide health facility survey conducted in PNG between April to November, 2012. This survey was independent to the pilot study described in this paper and was conducted according to the same methodology of an earlier country-wide health facility survey previously described in the literature [3].

### Data analysis

Data from the transmission reports, study diaries and complementary health facility survey were entered onto an Excel spreadsheet and descriptive statistics conducted as required. All GDs were recorded on a digital voice recorder, transcribed verbatim, translated into English, and entered into NVIVO 9. A thematic analysis of the interview data was conducted as informed by a general inductive methodology [20]. Interview data were independently coded by two investigators. Initial codes were derived from the research aims and were subsequently refined over two coding cycles. The two coders compared and agreed upon codes and emerging themes at the end of each cycle, resolving disagreement by consensus opinion or by the creation of new, mutually agreeable, codes/themes.

## Results

### Sample

A total of 42 health workers participated in the two-week pilot. Completed study diaries were obtained from 57% (24/42) of participating health workers. Reasons for not returning a study diary included: didn’t receive a study diary (n=9), didn’t complete the study diary (n=3), study diary was lost (n=2), participant was transferred or on leave prior to diary collection (n=2) and unknown (n=2). Six GDs, one in each site, were completed with a combined total of 17 participants.

### Transmission summary

Table 2 presents an overview of the text message transmission report as provided by the gateway company. A total of 792 text messages were transmitted during the pilot study of which 30% were delayed and 3% failed; failure was defined as a delay of 48 hours at which point the transmission was cancelled. Over half of the transmission failures (n=14) were the result of one participating health worker (from Sigerihi) relocating to an area with no network coverage for 7 days during the 10-day pilot. The median transmission delay, when delays occurred, was 2.43 hours.

**Table 2:** Overview of text message transmission.

**Study Site**	**Participants**	**Text message transmission**	**Transmission failure** ^a^	**Transmission delay**	**Median delay**	**Min/max delay**
	N	N	N (%)	N (%)	Hrs. Mins	Hrs. Mins
Kwongi	4	64^b^	8 (13)	39 (61)	2.04	0.01/47.22
Lopi	9	180	2 (1)	55 (31)	3.30	0.03/47.39
Yauna	6	120	0 (0)	31 (26)	3.28	0.01/46.55
Sigerehi	5	100	14 (14)	23 (23)	3.40	0.15/45.25
North	10	200	0 (0)	53 (27)	2.34	0.01/24.16
Lufa	8	128^b^	0 (0)	35 (27)	2.02	0.01/29.19
**Total**	**42**	**792**	**24 (3)**	**236 (30)**	**2.43**	**%1.1/47.39**

a. Failure = text message could not be delivered within 48 hours of original transmission. b. Transmission records were only available for 8/10 days.

### Study diary findings

Seventy-five percent (18/24) of participants who completed the study diary reported that their mobile phone was available to them at all times during the 10-day pilot. The remaining 25% (6/24) of participants reported that their mobile phones were not available for a median of 3 hours on 4.5/10 days. The primary reasons given for phone unavailability were visiting remote areas on a heath care patrol (n=10) and leaving the phone at home whilst at work (n=7). Twenty-nine percent (7/24) of participants reported that at least one of the text messages was received 30 minutes or more after the scheduled transmission time (transmission delay) and 13% (3/24) reported that at least one of the scheduled text messages was not received (transmission failure). Overall, the median delay between the reported time of receiving a text message and the reported time of reading that same message was 5 minutes (min 0 mins, max 619 mins).

### Group discussion findings

#### Network coverage and mobile phone access

Participants from rural health facilities frequently stated that the quality of network coverage varied depending on such factors as the weather, fuel supply at the transmission towers and proximity to the transmission towers. Nevertheless, even if network coverage couldn’t be guaranteed at a particular time or in a particular place, network coverage was generally available at some point within a 48 hour period or could be achieved by relocating to a known ‘good coverage’ spot. Participants from the urban health centres reported more reliable network coverage and, despite the disruptions that did occur, no participant expressed frustration with the quality of network coverage available or preference for an alternative network. All participants reported owning at least one mobile phone which, in all cases, was considered their own personal property for (typically) their own exclusive use. Participants usually carried their mobile phones with them at all times, including when at work, unless the phone was charging or had been forgotten:

“Mobile phone is like the clothes we are wearing. We carry mobile phone everywhere we go”

A small number of participants reported changing their SIM card within the past 12 months due to loss or theft, although many reported using the same phone and SIM for two or more years and others reported losing a phone, but having their old phone number transferred to a new SIM card.

#### Frequency and timing of text message transmission

The transmission schedule of two texts per day was widely acceptable, with only a small number of participants suggesting transmission should be more or less frequent. The morning text message was generally considered most acceptable as it was a time when participants were alert and at work, enabling them to put the information in the received text into immediate practice:

“I think in the morning it’s the right time [to receive the text messages] and not in the afternoon because that’s the time we are with the patient and we might need the information to help our patients.”

Receiving text messages late in the afternoon or after hours (due to delays in transmission) was often considered less appealing as participants were no longer in the work environment at that time and were pre-occupied with other tasks:

When you text while we are at work it reminds us so that’s good. After hours we are already at home with our families so we won’t bother reading the text (Participant 1, Segerihi).

The repetition of the 10 text messages (twice) over the two week pilot period drew a wider range of comment. The repetition was generally considered helpful as it reinforced the intended message; however, a number of participants suggested continued repetition of the same messages beyond the two week pilot period would be of little appeal:

“once or twice is enough because the more we read the same text message the more we lose interest in reading it.”

Participants in one GD recommended halting transmission for a two to three month period and then repeating the two week transmission schedule. It was also frequently suggested that new messages should be included in the transmission schedule to keep the content fresh and stimulating

“The first round of messages come and go, but the next round we should have new messages”

#### Text message access

All GD participants received at least one text message during the two week pilot period, although a number noted delays in transmission: “Sometimes I received three messages [in one day] because of the network coverage; the text from the previous day also comes in.” (*Participant 1, Segerihi*). Other barriers to regular text message access were reported, including switching the phone off to preserve the battery charge: “I live in a very remote area and it’s difficult to charge my mobile phones so when we want to make calls we usually switch on our phones.” (*Participant 2, Segerihi*); unavailability when charging the battery: “If my battery is low then I have to charge it or look for other places to charge it then I don’t read the text messages on time, but later” (*Participant 1, Kwongi*); and in one case, language difficulties: “I find it difficult to read the text myself so I give it to someone to read for me” (*Participant 2, Segerihi*). Nevertheless, most participants reported receiving all or most of the transmitted text messages on a regular basis.

Text messages were typically accessed at work, either during breaks or, if time permitted, at the time of transmission: “when I am not busy with the patients and I receive a text message I read it on the spot, but when I’m busy I leave it and read it when I am free.” (*Participant 2, Yauna*). A number of participants reported that they read each message more than once, “I read it two or three times to really understand the text message” (*Participant 2, Lufa*), and many stated that they discussed or shared the content of the text messages with their colleagues. For example, the officer in charge of one of the participating health facilities reported doing the following upon receiving a text message: “*In the morning, as I receive my message I tell them* [*the health facility staff*] *that the people out there* [*the senders of the text message*] *have said you must not do this and that.* “Don’t give MALA 1 to pregnant women in their first trimester” *[the content of one of the ten text messages*]*, are you all aware of this? I tell them and then go to my office* (*Participant 4, Lopi*).

#### Text message content

Participating health workers overwhelmingly expressed a preference for English language text messages, often stating that *Tok Pisin* translations would be lengthy and open to misinterpretation: “Mostly for us health personnel, we speak pidgin everyday with the patients and so we are good at it; however, it takes a long time to read a pidgin word. With English we can’t speak it, but can read and write it fluently. Thus, English is okay (Participant 3, Lopi).”. However, it was noted that English language texts may be difficult for elderly health workers stationed in isolated settings to understand. Similarly, with the exception of the one participant who found it difficult to read the English language text messages, all other participants reported that the wording of the ten text messages was clear, easy to understand and appropriate for health workers: “The wording of the text messages is in the language of medical professionals like us and we understand it. It’s straight forward so there’s no need for changes” (*Participant 1, Lopi*). When asked, which of the ten text messages were most useful, responses such as “I think all the text messages are useful” (*Participant 1, North*) were common. Stated preferences varied widely among participants when subsequently pressed. No participant suggested any of the messages were unhelpful or unnecessary, although in some cases the messages presented new information to participants instead of serving as a reminder about information already imparted. In these cases, the participants often had questions following text message transmission. For example: “In the case of [text message number] seven, “prescribe MALA 1 to all patients with a positive RDT/mps, unless patient is in first trimester of pregnancy”, what would we do or give to women in such condition?” (*Participant 2, Lopi*)*.*

#### Perceived utility of the text message reminder service

Without exception, all GD participants reported that the pilot text message reminder service was helpful to their clinical practice. The following excerpt was typical in this regard: “The text messages received reminds us on the steps to take when treating a fever patient from testing to diagnosis and also on what advice we will give to the patients. It was very helpful” (*Participant 3, Kwongi*). A number of participants stated that a text message service of this type would be especially beneficial in rural parts of PNG where health workers often work in isolation and are rarely able to participate in formal training or continuing education opportunities. The simplicity of the text message service also held considerable appeal as it did not require much effort on the health workers behalf to receive the information. Many participants favourably compared the ease of the text message intervention with other, more intensive, sources of information:

“Many of us went through the short refresher courses [providing instruction on the new NMTP] and we came out with pamphlets and books. If you go into a health worker’s house it’s filled with books, but we don’t take time reading those books. Receiving text messages in our phones is a very helpful method of reminding us and refreshing us every day. We haven’t read books in a while and we’ve forgotten how to read, but we are good at reading text messages. It’s very useful. If we keep on reading these text messages and others we will be experts in our fields.”

The experience of easily receiving clinical instruction during work hours, when the health workers were treating patients, also held appeal as succinctly expressed in the following excerpt: “Our booklets are at home and our mobile phone is in the pocket so when the text message comes it reminds us so we work fast and treat fever cases” (*Participant 1, Sigerehi*). The acceptability of the pilot intervention was further demonstrated by an expressed interest among study participants to extend the service to include other illnesses: “The text message reminder [service] is a really good initiative [the Papua New Guinea Institute of Medical Research] is doing for malaria control and it’s helping us, but I am wondering if this approach can be done with other diseases? Because with STDs [sexually transmitted disease] it’s hard to differentiate signs and symptoms and text reminders will help us to treat and that goes for TB [tuberculosis] too.” (*Participant 1, Kwongi*)*.*

### Health facility survey data

A total of 212 health worker interviews were completed during the countrywide health facility survey. Of these 212 health workers, 92% (195/212) reported owning at least one mobile phone. Of these 195 health workers, 99% (193/195) were connected to the Digicel network, 8% (15/195) to the B-Mobile network and <1% (1/195) to the Citifon network.

## Discussion

The findings presented above indicate that a text message reminder service to support health workers implement the new NMTP in PNG is both feasible and acceptable. Data from the transmission report, study diaries and GDs suggest that, whilst delays were not uncommon, the vast majority of text messages were successfully transmitted. Similarly, participants’ mobile phone access was generally regular enough to ensure that most text messages were read most of the time, typically shortly after they were received and often in the clinical environment (although a small number of participants reported that they lost their mobile phone during the course of the pilot, this was not common and new SIM cards with the old phone number could be purchased). Thus, network coverage and mobile phone access did not present as major barriers to intervention implementation.

The text message reminder service itself was highly regarded. The transmission schedule of two messages per day was well received, although a clear preference for text messages delivered in the mornings and during work hours was evident. The repetition of the 10 text messages over a two week period was also acceptable; nevertheless, it was notable that many participants expressed antipathy towards the prospect of continued repetition of the same messages. Almost all participants reported that the 10 text messages were easily understood and clinically useful and no one message was widely considered more or less useful than the others. One participant reported the English language text messages difficult to understand, but this was the only criticism of the content and English was still widely considered the most appropriate language for mobile phone-based communication to health workers. The pilot intervention compared favorably to in-person training and other resources previously provided in support of the new NMTP. The ease with which the text-based information was imparted and the fact that the text messages were typically delivered within a ‘real world’ clinical context were particularly appealing. The acceptability of the intervention was further highlighted by repeated requests for the intervention to be applied to other commonly occurring diseases.

In addition to the largely positive pilot outcomes, complementary data from a country-wide health facility survey suggest that a majority of health workers in PNG have access to a mobile phone and that the vast majority of these health workers are accessible via a single network provider; the provider used in this pilot. The conditions exist, therefore, to implement the text message reminder service on a far broader scale than was attempted herein. Nevertheless, the study findings highlight a number of issues that warrant careful consideration before any such implementation should take place. The first issue pertains to the optimal number of times the text messages should be repeated. In the study upon which this pilot was based, the same 10 text messages were repeated weekly over a 26 week period [16]. Participant responses indicate a single topic text message ‘cycle’ of this duration would be too extensive in PNG; rather, a more locally appropriate model may be to cycle a set of malaria-related text messages over a briefer time period (e.g. 4-8 weeks) and then introduce a new set of text messages in another disease area (e.g. the treatment of pneumonia, STIs or non-malarial fevers) in a second cycle of equal duration. This approach is consistent with recent calls to trial mhealth interventions that support health workers to better manage a range of commonly occurring diseases [18] and would further inform the evidence base as to what number of message repetitions are required to affect a change in health worker practice.

Secondly, the response of some GD participants was suggestive of ambivalence towards the new NMTP. For example, NMTP-related manuals and job aides were reportedly received, but not utilized and when text message ‘reminders’ raised follow-up questions (e.g. what to prescribe women in the first trimester of pregnancy who test positive for malaria parasitaemia) few (if any) participants thought to consult these resources. Undoubtedly, the text message-based intervention utilized in this pilot had appeal because its uptake required little effort on the participants’ behalf, yet even a relatively user-friendly intervention such as this may have limited impact on health worker practice in the face of ambivalence towards the desired change. The relative ease of the text message reminder service may even discourage use of complementary – yet more complex – resources such as a written treatment protocol if delivered in a context of ambivalence. This is potentially problematic as the piloted intervention was designed to remind participants of knowledge that should already have been imparted and is insufficient as a primary source of new information.

Behavior change processes among health workers faced with new or revised treatment protocols have not been widely studied, especially in low-to-middle income settings [15,21]. The Kenyan trial of a text message reminder service to support the implementation of a new NMTP found some evidence that the resulting behavior change was consistent with Prochaksa and DiClemente’s stages of change model [17], yet further study is required in this area. Accordingly, any scale up or future trial of a text message reminder service to support a change in health worker practice should incorporate a detailed analysis of the behavior change process observed in the face of the intervention and/or a proportion of the text messages could be specifically designed to promote improved practice according to existing models of behavior change. In the context of this study an example might have included the following text message: “did you know that most fever patients in PNG test negative for malaria infection when tested by RDT or mps”. In a stages of change model, a message such as this could potentially promote a shift from a ‘precontemplative’ stage (in which the health worker is not even considering the importance or necessity of the change in NMTP) to a ‘contemplative’ stage in which he/she actively contemplates the pros and cons of protocol change and, as such, moves closer to the ‘action’ stage in which he/she actively engages in a change process [22]. Inclusion of text message reminders encouraging the use of existing, more detailed, resources (e.g. a written treatment protocol) may also further improve health worker performance and limit the risk of the text messages becoming a primary source of knowledge.

Finally, despite the apparent success of this study, the evidence base to support scale up of the piloted intervention is limited. The ability of a text message reminder service to facilitate a sustained and clinically meaningful change in health worker practice has yet to be demonstrated via rigorous research inquiry [21,23]. Similarly, important questions such as the optimal intervention duration have yet to be adequately addressed. Given these limitations in the evidence base it would be premature to advocate a nationwide scale up of the pilot text message reminder service in PNG. Rather, a more limited scale up of the pilot intervention in the context of a robust research project, similar to the cluster randomized trial conducted in Kenya, is a logical next step. Ideally, any such trial would be conducted in the more highly malaria endemic provinces of PNG and should seek to incorporate some of the recommendations resulting from the pilot study described in this paper; in particular, the use of multiple, but relatively brief text message cycles variously focusing on the treatment of malaria and other common diseases, with the incorporation of theory-based promoters of behavior change as well as a detailed investigation of the behavior change process incorporated into the study design. The resulting findings would not only better inform policy makers re the potential impact of scaling up such an intervention in PNG, they would also usefully address major gaps in the international evidence-base pertaining to the use of text message reminders to support improved health worker practice.

There were a number of limitations in our study. First, the pilot was only conducted in one of the 22 provinces of Papua New Guinea and, as such, network coverage issues identified in the context of this study may or may not be reflective of potential issues in other parts of the country. Network coverage is rapidly improving across PNG and mobile phone ownership continues to increase. Any network coverage and mobile phone access issues relevant in other parts of the country, therefore, are likely to decrease over time, at least in the short-to-mid-term. Second, study diaries were not obtained from a large proportion of participants, raising the possibility of non-response bias. Third, fewer than half of the study participants participated in the GDs. Nevertheless, the findings from the various data sources were consistent and data saturation was achieved among those who did participate in the GDs. This would suggest that the findings reflect the general experience of the participants included in the pilot. Thus, on the basis of this study, we conclude that a text message reminder service designed to support PNG health workers to implement the new NMTP is both feasible and acceptable. A pragmatic randomised controlled trial incorporating the recommendations made from the current study is needed to provide the robust evidence of effectiveness required prior to consideration of widespread scale up.
